# Management of facial burns

**DOI:** 10.1093/burnst/tkaa023

**Published:** 2020-07-06

**Authors:** David G Greenhalgh

**Affiliations:** 1 Burn Department, Shriners Hospitals for Children Northern California, 2425 Stockton Blvd., Sacramento, California, 95817, USA; 2 Firefighters Regional Burn Center at University of California, Davis, Department of Surgery, University of California, Davis, 2315 Stockton Blvd., Sacramento, California, 95817, USA

**Keywords:** Burns, Scar, Face, Skin grafts, Contracture, Esthetics

## Abstract

Burns to the face affect a part of the body that cannot be hidden and thus exposes potentially major changes in appearance to society. Therefore, it is incumbent upon the caregiver to optimize healing and minimize scarring. The goal for partial-thickness burns is to have them heal within 2–3 weeks to minimize healing time. For full-thickness burns there needs to be strategies to optimize the outcomes for skin grafting and minimize scarring. The goal of this review is to discuss the best way to improve the outcomes of these devastating injuries.

## Background

Our faces identify us and are always exposed to the public. Huge amounts of money are spent on makeup and cosmetic surgery in order to improve appearance. Any facial defect is immediately noticed and leads to stigmatization by society. Burns to the face are particularly noticeable and may lead to lifelong changes in appearance. The goal of this paper is to review the unique characteristics of facial burns and describe techniques to optimize their functional and cosmetic outcomes.

## Review

### Unique aspects of face burns

The face is at a higher risk of burn injury because it is rarely covered by clothing or other protective gear. Therefore, any exposure to heat in the vicinity of the face may cause a burn. For instance, flash burns, such as those from explosive fumes (propane, natural gas or butane) will burn any exposed skin but spare those covered with clothing. Spilled liquid from high places will reach the head and face of children. Fortunately, the face has some protective characteristics that can reduce the depth of injury. Four factors are classically described to determine the depth of a burn injury. The first, the temperature of the inciting agent, cannot be altered. The second, duration of contact, can be reduced since people tend to protect their faces by shaking or wiping off any hot material as soon as possible. In addition, any material that absorbs the heat and stays in contact with the skin will deepen the burn. Since there is rarely any clothing on the face, duration of contact is minimized. The third factor is the thickness of the skin. Face skin is relatively thick in most parts, except for the eyelids, and thus burns are not usually as deep as thinner areas. The fourth factor is blood supply of the skin. The more blood supply, the greater the ability to dissipate heat and reduce the depth of injury. The face is extremely vascular so that heat is dissipated to reduce severity of injury. The increased blood supply should also be kept in mind when excising the face since bleeding exceeds that of any other area of the body.

There are some anatomic issues that affect the ultimate outcomes of face burns. The forehead skin sits mostly on bone and has little flexibility. Burns to the forehead tend to contract less than burns in other areas. Deep burns may lead to exposed skull, which becomes a very difficult problem. The remainder of the face sits on fat and muscle and is highly mobile. Any small but deep burn in these areas tends to shrink with little resistance and is more prone to contractures. This problem is particularly important for eyelids and lips since they provide little resistance to the forces of contraction. The eyelids are prone to forming ectropions, and shrinkage of the commissures or eversion of the lips is not uncommon. Even small burns such as those seen after chewing an electric cord that leads to a commissure burn leave permanent scars. Since the head is usually flexed or at least in a neutral position, neck contractures are another common problem. Contractures are more of a problem in children since their skin lacks laxity. With aging, the skin loosens and thus is more tolerant of shrinkage. Reducing this laxity is the premise of face lifts or, even more relevant, the “controlled” burn of a chemical peel. Some parts of the face have very little fat, are very thin and prone to contractures. The nose skin, lying on cartilage and bone, has very little buffer to prevent exposure of these ungraftable structures. Contractures of the nasal alar and shrinkage of the nostrils are difficult problems. The skin of the external ear sits on cartilage so that any third-degree burn may lead to cartilage exposure and predispose this relatively avascular area to infection (chondritis). Deformity of burned ears tends to be common. It is not possible to excise ears since cartilage would be exposed and grafts will not vascularize. One must wait for granulation to form before any grafting attempt.

### Society and the burned face

Our identity in in society is dictated by our face. Attractive faces are admired and regular faces are accepted. Any deviation from normal leads to immediate notice from the public and, often, stares and questions. Even polite people will stare and, with good intentions, ask what happened. Children tend to feel that there is something wrong with the scarred person and either cry or taunt the person. It is a sad statement that facial deformity is associated with being evil in our society. This persona has been portrayed by many of movies or television shows. The Phoenix Society reported a review of 32 films produced between 1933 and 2017 that portrayed burn survivors in a very negative light [1]. Almost all characters were isolated from the other characters and only five had friends. Half of the characters were “good” before injury but became “evil” after their injuries. None of the movies had happy endings and only four had “hopeful” endings. The majority (68.7%) were ashamed of their scars, 15.6% had any friends and 62.5% were out for revenge. Like many actual burn survivors, 68.7% hid their scars, but, to make matters worse, revealing their scars was the “plot point” (or crisis point) of the movie. In the end, 40.6% died. These movies have a profound impact on burn survivors. I have had more than one patient tell me that they “did not want to end up looking like Freddy Krueger”. Our goal, then, is to ensure that the outcomes of facial burns are as good as possible.

### Optimizing cosmetic outcomes in facial burns

The goal of treatment of facial burns is to do the best to allow the patient’s appearance to return to as normal as possible. Clearly, the ultimate outcome depends on the severity of injury. The deeper the burn and the larger the total body surface area (TBSA) involved, the more difficult it is to end up with a face that appears normal. Other factors influence the extent of scarring for any burn. Older people tend to have looser skin so they can tolerate contraction better, but they may have donor sites that have more problems with healing. Genetic factors are also important, since some patients tend to scar more than others. In the author’s experience, people from Southeast Asia tend to scar more than other groups, but it is difficult to predict whether an individual will have that tendency. Finally, compliance with physical and occupational therapy makes a huge difference to the ultimate outcome. This review will cover the management of facial burns, beginning with the least severe.

### Management of partial-thickness burns of the face

**Figure 1.**
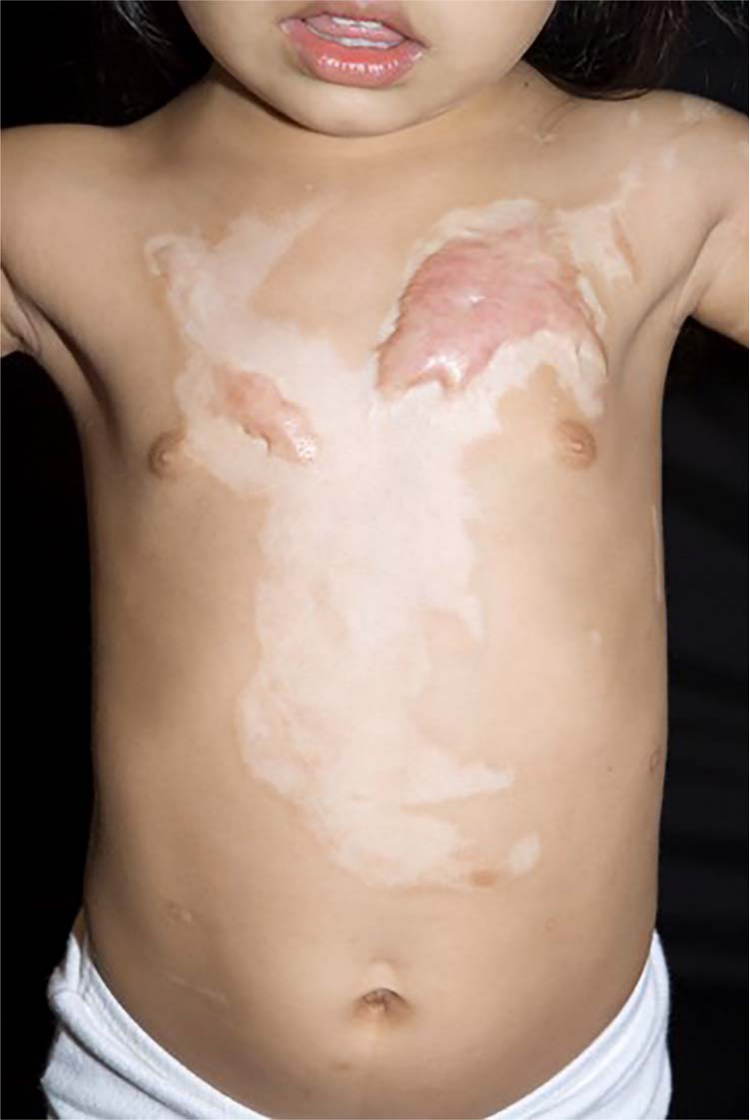
In the middle of a flat healed burn, one area has become hypertrophic because it took a few days longer to re-epithelialize

Since the dermis and its adnexa are intact, partial-thickness burns heal by re-epithelialization. In the 1980s, Deitch published data that suggested that a delay in re-epithelialization by 2–3 weeks increased the risk for hypertrophic scarring [2]. Since that time, all burn clinicians have experienced hypertrophic scars in wounds that have delayed wound closure. The remarkable finding is that within the same wound some areas will be perfectly flat but other areas that healed a few days later will develop hypertrophic scarring (Figure 1). The signals that induce these changes based on time are unknown. The goal for any partial-thickness wound is to have it heal in under 2–3 weeks. There are several ways to improve the rate of re-epithelialization. First, the new epithelium travels faster in a moist environment than when allowed to dry. If allowed to dry, the exudate from the wound forms a scab that requires removal by the epithelial cells with proteases before they migrate across the wound surface. This process delays closure and increases the chance of hypertrophic scarring. Ointments that maintain the moist environment work very well. There are some biologic dressings that adhere to the wound and maintain an optimal moist environment, but they are difficult to maintain on the face and thus are rarely used.

Avoiding infections will also reduce the chance for hypertrophic scarring. The face should be washed with soap and water at least once a day. Antimicrobial ointments are effective and require reapplication when they dry up. One of our favorites is bacitracin, which covers gram-positive organisms. We apply it 4–6 times per day, depending on whether it disappears or not. One should not use bacitracin for longer than a week or so, since a rash (probably from yeast overgrowth) often occurs. Other ointments either with or without antimicrobials may be used instead of bacitracin. One should not use silver sulfadiazine since it tends to impair re-epithelialization and thus may increase the risks of scarring [3].

### Treatment of deep face burns with skin grafting

If the face burns are still open at 2–3 weeks, then one must make a decision as to whether skin grafting is needed. There are several matters that should be considered before skin grafting the face. First of all, one should assess the size of the area and how the burn fits into the esthetic units of the face [4]. When one looks at the face, there are components that that are considered as “whole” and should not be violated. If the esthetic unit has a scar or lesion it is noticeable to others. Esthetic units include the entire forehead, cheeks, nose, lips and chin. Scars at the junctions of these units are much less noticeable, so the goal of any surgery is to place incisions, or skin graft seams at the junctions. For instance, plastic surgeons make facelift incisions at the hairline (and not in the middle of the cheek). If possible, skin grafts should follow the same principles. In addition, small grafts in the middle of these units tend to be just as noticeable as a scar. Another consideration is the facial site where a graft is required. The forehead has skin that is relatively immobile and exists over the firm skull. Grafts on the forehead tend to do better than other areas since pressure works better and the ability to contract is more limited. Skin grafts on the cheeks and lower face are placed on much more mobile tissue that is not directly over bone so there is a greater tendency for these to contract in comparison to the forehead. The decision to graft a small isolated third-degree burn of the cheek is difficult because both choices, graft or healed scar, are very noticeable and ugly.

Once the decision to graft the face is made there are some important issues that the surgical team must consider. Faces are very vascular, so they bleed significantly when excised. The rule of thumb for blood loss for excision and grafting for all areas, except the face, is around 2% of blood volume per percent of TBSA excised [5]. So, if one is going to excise and graft a 25% TBSA burn, then one should prepare for the loss of 50% of total blood volume. Faces tend to bleed around 4.5% of blood volume per percent of TBSA excised. If one is going to excise a child’s face (around 10%) then one should expect to lose 45% of that child’s blood volume. Clearly, one must ensure that there is enough blood available for transfusion during the procedure. To reduce blood loss, we always inject lactated Ringer’s solution that contains 2 mg/L of epinephrine beneath the burn. The epinephrine significantly reduces bleeding from small vessels. Another important point is to excise the burn to viable tissue rapidly, as opposed to taking a slice, obtaining hemostasis and then re-excising the same area a second or third time. Communication with the anesthesiologist is important to ensure the patient does not fall behind in resuscitation.

**Figure 2.**
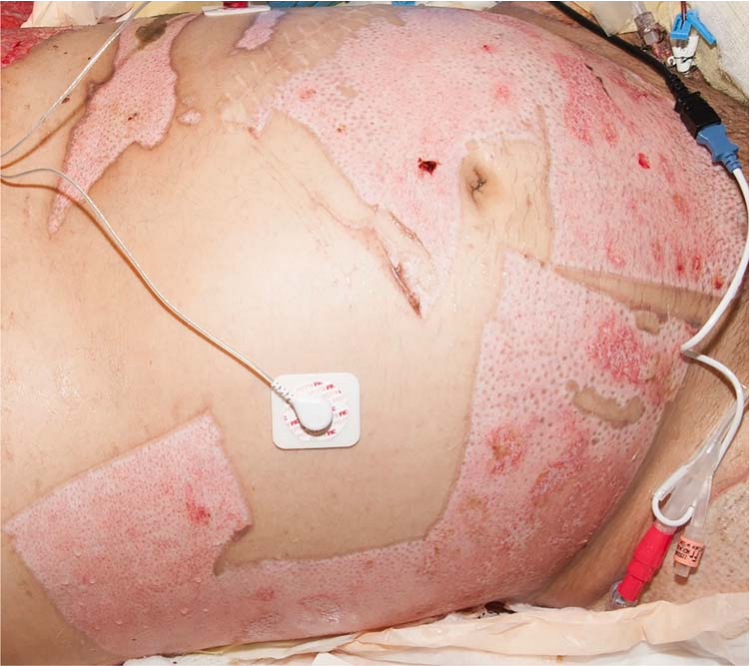
This patient had two “U-shaped” split-thickness donor site harvests that were designed to match the recipient site of the face. One piece was placed on the top of his head and the other on the lower face

**Figure 3.**
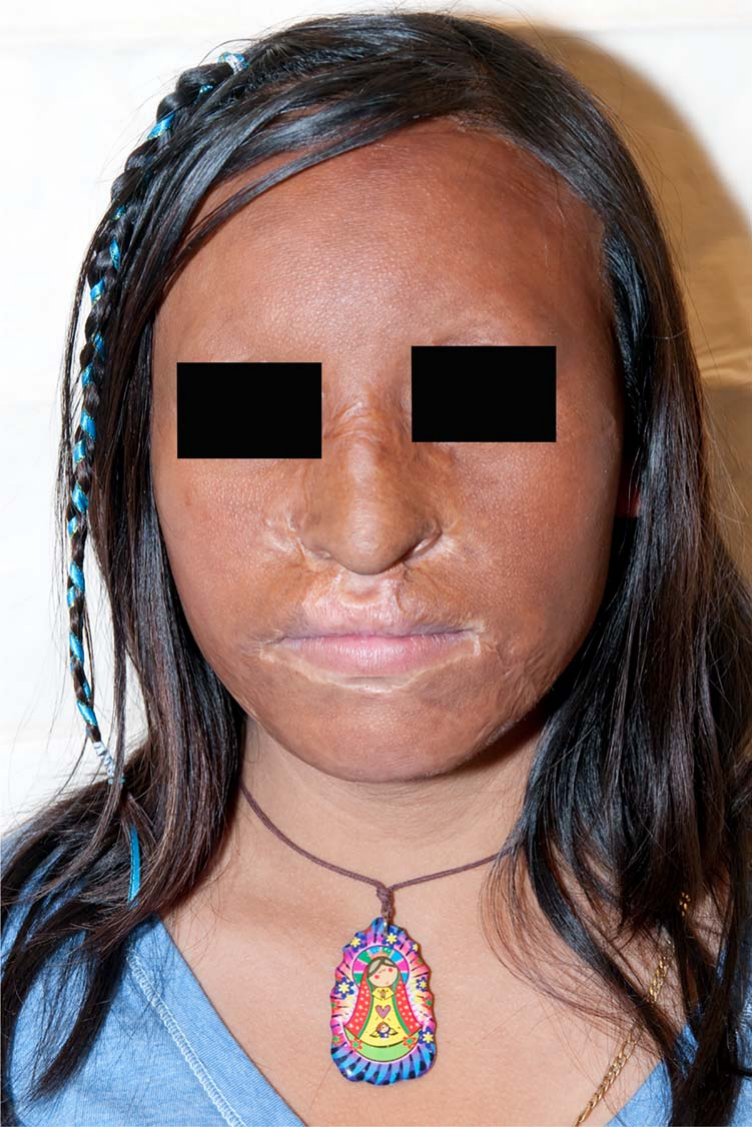
A female patient more than 1 year post-third-degree burns to the face had an “U-shaped” autograft which demonstrates minimal scars from the seams

Another consideration is the location of the donor site. Skin harvested from below the clavicle has a darker pigmentation than skin from above the clavicle. The scalp is the best choice to avoid an obvious color difference. Scalp skin is more difficult to harvest, and one must try to eliminate as much hair (by scraping the dermal side) as possible. Otherwise it is not uncommon to have some hair transferred with the graft. If the entire face is involved there will be no obvious color mismatch so the largest and best-quality donor site should be harvested. The author has developed a technique to harvest skin in a circular or “U-shape” that can be wrapped around the face (Figure 2 and 3) [6]. The goal is to match the donor skin with the shape of the recipient site, while at the same time minimizing the number of seams between the edges of skin graft. The availability of a 6-inch-wide dermatome makes the possibility of covering a face with one piece of skin easier. The goal is to cover the cheeks and forehead with one piece of skin. The face graft should never be meshed since the mesh pattern will exist for the rest of the patient’s life.

**Figure 4.**
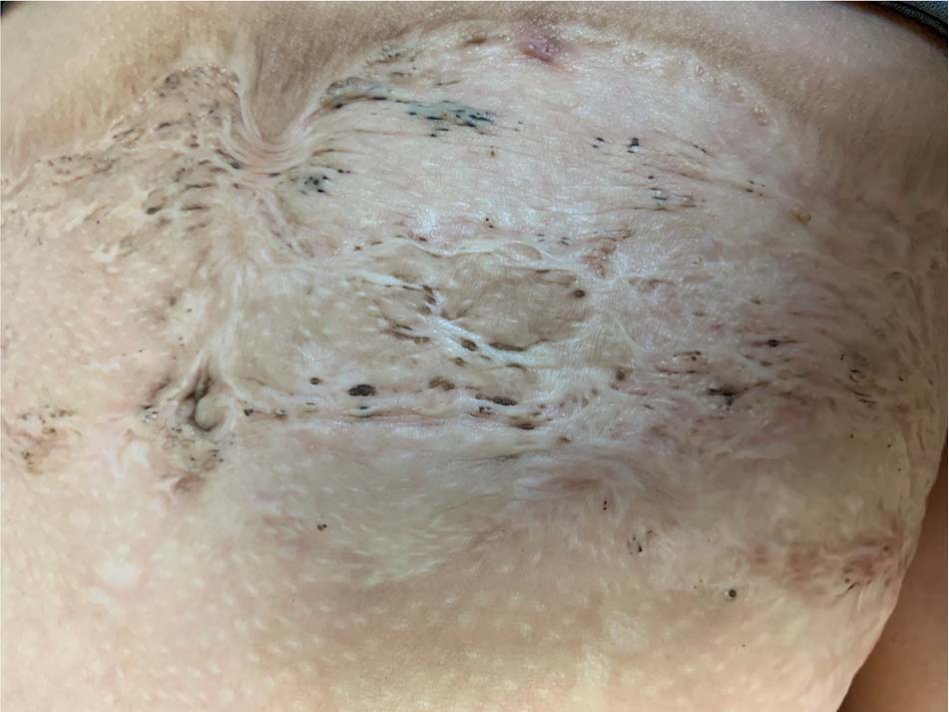
The appearance of “sponge skin” that is the result of incompletely excised dermis from the wound bed. There are healed areas that create skin bridges which collect dirt and give the appearance of “black heads”

The most common cause of graft loss after a face graft is from hematomas. We leave the face graft open to air so that any hematomas can be seen and drained. Initially, hematomas can be “rolled” to the edge of the graft, but after a day the graft adheres to the wound bed so rolling will lift the adherent graft. After the first day, we make a nick in the graft to drain the hematoma. A decision needs to be made at the time of facial burn excision as to whether to immediately place an autograft or to apply a temporary wound coverage. If hemostasis appears adequate, immediate coverage with autograft works fine. If there seems to be too much bleeding, then applying an allograft or a dermal substitute is appropriate. The philosophy that grafting on a dermal substitute reduces scarring is not correct since we have had excellent results with immediate grafting on fat. It is essential, however, that all dermal elements are removed to prevent the development of inclusion cysts or skin bridges, called “sponge skin” (Figure 4) [7].

One must adjust the plan when dealing with a full-thickness face burn in a patient with a large TBSA burn. In a massive burn, there is some debate as to whether the face should take priority over other body parts. We will excise and autograft hands and arms first, followed by excising and temporary coverage of burns of the legs and trunk as a primary procedure. We typically excise the face as a separate procedure but will do so early (within a week) in the course of treatment. There are several reasons for delaying the treatment of the face. First, the depth of injury to the face is often not as severe as other areas, and so parts of the face may heal on their own. In addition, with a major burn, the luxury of harvesting a thick donor is lost since the goal is to reharvest the skin multiple times. The use of dermal substitutes as temporary coverage allows for the use of a thinner donor and may reduce the ultimate scarring.

**Figure 5.**
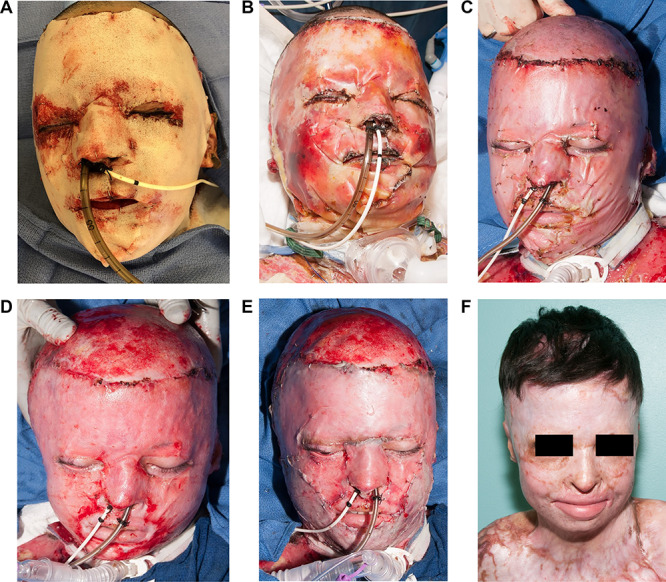
A series of pictures demonstrate the placement of BTM™ on a patient with 90% total body surface area burns. **(a)** At the time of placement of the BTM™. **(b)** Postoperative day 2. **(c)** On day 73 the BTM™ is well-vascularized and has had no infections. **(d)** On the same day, the outer matrix was delaminated. **(e)** The surface was covered with his available skin. We did not have enough skin to maintain esthetic units. **(f)** The appearance of the face more than a year after the burn

If there is significant risk for hematoma formation, we will cover the excised face with allograft for 1–2 days and then replace the allograft with a dermal substitute. There are several dermal matrices available for use on the face. In the United States, the two most commonly used matrices are Integra™ (Integra LifeSciences, USA) and Biodegradable Temporizing Matrix™ (BTM™) (Polynovo Biomaterials Pty. Ltd., Australia). Integra™ is a collagen type I matrix that is covered with a silicone surface. Inflammatory and endothelial cells from the wound migrate into the collagen matrix to replace it and create a vascular bed that will accept a thin autograft. The silicone acts as a barrier to outside bacterial invasion to reduce excessive inflammatory response and minimize granulation tissue formation. The process takes a minimum of 2–3 weeks before the silicone can be removed and autografted. If all goes well, the dermal matrix reduces scarring after being covered with a thin autograft. There is a tendency for pockets of purulence to form under the silicone and, if not controlled with incision and drainage, infection can spread to destroy the entire matrix. BTM™ follows the same principles but, instead of being a matrix of collagen, the dermal component is a polyurethane foam and the outer protective surface is a polyurethane sheet. The same cells from the wound invade the foam and gradually replace it. The outer sheet tends to suppress inflammation and granulation tissue formation. Like Integra™, BTM™ requires a minimum of 2–3 weeks before it has vascularized enough to accept an autograft. At that point the outer polyurethane sheet is removed and covered with a thin autograft. BTM™ seems to be more resistant to infection compared to any other dermal substitute and seems to minimize the inflammatory response. We have been able to leave the BTM™ on the face for several weeks and have had excellent results (Figure 5).

**Figure 6.**
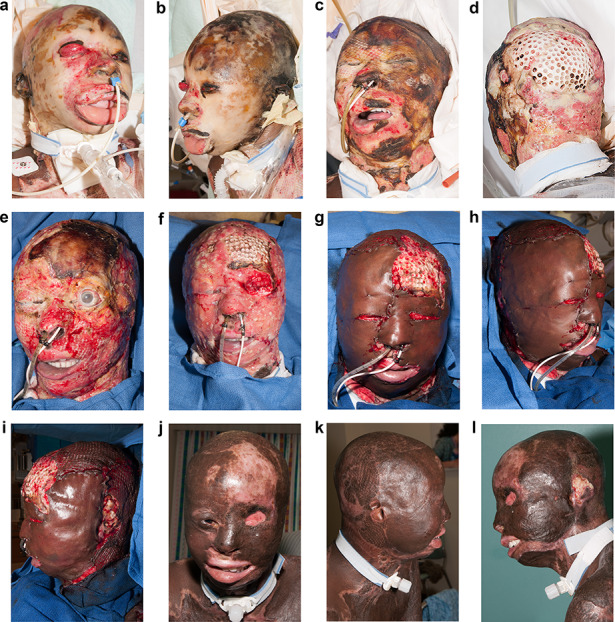
A series of pictures of a patient with fourth-degree burns to her face. The burn was caused by burning her hair extensions, which ignited and the patient was unable to extinguish the flames. **(a–b)** The burn on admission. **(c)** The burns were excised and covered with allograft. It is clear that the frontal region is not viable. **(d)** The outer table of the occipital skull has been burred to induce granulation tissue. **(e)** The left eyelids were lost, and she eventually lost her left eye. **(f)** The face was covered with Integra™. Image shows the Integra on the day of delamination—it was vascularized with only small pockets of infection. **(g–i)** The viable wounds were covered with the same principle of minimizing seams. The 6-inch dermatome was used. **(j–l)** The face approximately 6 months after autografting

The ultimate outcome of a face burn is dependent on the severity of injury and the availability of good donor sites. We have had some patients with fourth-degree burns to the face and scalp. As an example, we had a patient who was burning her hair extensions, which ignited and she sustained deep burns to the upper torso and face, with ultimate loss of her left eyelids and exposure of the bone of the scalp and forehead (Figure 6). Since we were not sure about the viability of the wound bed, we initially excised the burn and covered it with allograft. Since there was no viable tissue for a local flap, the exposed skull had multiple holes burred into the outer table to encourage granulation tissue formation. The left eyelids were not viable and, despite our efforts, she lost her left eye. Eventually, we covered her face with Integra™ and, when we had adequate vascularity, we were able to harvest a large U-shaped piece of skin to give her a presentable autograft. The ultimate outcome was as good as possible.

### Scar management of face burns

**Figure 7.**
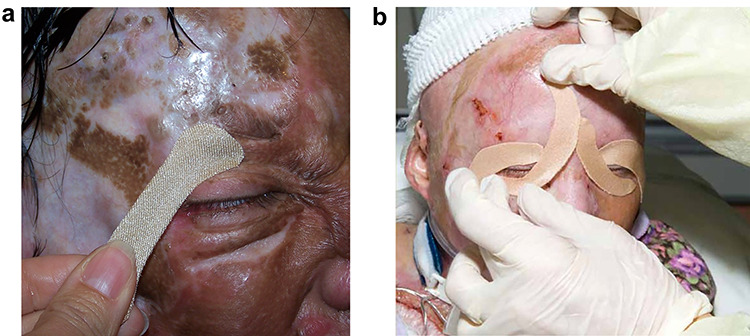
The use of silicone tape to reduce the formation of ectropions on a patient with deep face burns. **(a)** A tape is used to pull the upper eyelid down. **(b)** Both upper and lower traction tapes are applied

Scar management of face burns is essential but quite difficult. The role of an occupational or physical therapist is extremely important. While the forehead is relatively easy to deal with, the rest of the face has little resistance to contracture. Eyelids have little resistance to contracture and are often at risk for developing ectropions. The mouth and lips are also problem areas. The lips will often evert, and the size of mouth opening is often reduced.[8] Stretching and scar massage are helpful. We also will utilize both soft and hard facemasks to reduce scarring. In addition, we utilize silicone patches during the early phases of graft healing to reduce scarring. A novel idea has been to use silicone-based tapes to pull eyelids together in order to reduce ectropion formation (Figure 7). The entire scarring process persists for up to a year, so persistence with the therapy plan will lead to the best outcome. Despite the efforts of burn surgeons, the outcomes after face burns still lead to lifelong hurdles in survivors [9–11]. Our goal should be to continue to develop techniques to improve the outcomes of these injuries.

### Future management of face burns

There have been very little advances in the management of face burns over the past 40–50 years. The concept that one must enhance re-epithelialization in order to reduce scar formation has been around since the 1970s. There have been attempts (since the 1980s) to accelerate the rate of epithelial migration with bioengineered growth factors but they have not come to fruition. Hopefully, products that accelerate the resurfacing of partial-thickness wounds, such as autologous spray epithelial cells (e.g. RECELL®, Avita Medical, USA) will be improved. A preliminary study using these spray cells was encouraging [12]. For full-thickness burns the development of better grafts that are rapidly available and which reduce scarring is desirable. The technology for growing a composite skin consisting of autologous cells from the patient that create dermal and epidermal skin has been around for a while [13]. It would be a great advance if composite skins were available “off the shelf”. In order to accomplish that goal we will need to be able to eliminate the rejection of foreign cells.

## Conclusions

The treatment of face burns involves specialized care. The goal for partial-thickness burns is to optimize re-epithelialization in order to have them heal within 2 weeks. Deeper burns require skin grafting. There are techniques that improve the outcomes of skin grafts for face burns. Finally, occupational and physical therapy is essential in order to optimize the final outcome. We still have a long way to go to reconstruct a deeply burned face back to an acceptable outcome.

## Data Availability

This is a review based on experience and references.
